# Bone Fracture Pre-Ischemic Stroke Exacerbates Ischemic Cerebral Injury in Mice

**DOI:** 10.1371/journal.pone.0153835

**Published:** 2016-04-18

**Authors:** Liang Wang, Shuai Kang, Dingquan Zou, Lei Zhan, Zhengxi Li, Wan Zhu, Hua Su

**Affiliations:** 1 Department of Anesthesia and Perioperative Care, Center for Cerebrovascular Research, University of California San Francisco, San Francisco, California, United States of America; 2 Department of Neurosurgery, Tianjin Fifth Center Hospital, Tianjin, China; 3 Department of Neurosurgery, Beijing Tiantan Hospital, Beijing, China; 4 Department of Neurology, Shanghai Ninth People’s Hospital, Shanghai, China; Indian Institute of Integrative Medicine, INDIA

## Abstract

Ischemic stroke is a devastating complication of bone fracture. Bone fracture shortly after stroke enhances stroke injury by augmenting inflammation. We hypothesize that bone fracture shortly before ischemic stroke also exacerbates ischemic cerebral injury. Tibia fracture was performed 6 or 24 hours before permanent middle cerebral artery occlusion (pMCAO) on C57BL/6J mice or Ccr2^RFP/+^Cx3cr1^GFP/+^ mice that have the RFP gene knocked into one allele of Ccr2 gene and GFP gene knocked into one allele of Cx3cr1 gene. Behavior was tested 3 days after pMCAO. Infarct volume, the number of CD68^+^ cells, apoptotic neurons, bone marrow-derived macrophages (RFP^+^), and microgila (GFP^+^) in the peri-infarct region were quantified. Compared to mice subjected to pMCAO only, bone fracture 6 or 24 hours before pMCAO increased behavioral deficits, the infarct volume, and the number of CD68^+^ cells and apoptotic neurons in the peri-infarct area. Both bone marrow-derived macrophages (CCR2^+^) and microglia (CX3CR1^+^) increased in the peri-infarct regions of mice subjected to bone fracture before pMCAO compared to stroke-only mice. The mice subjected to bone fracture 6 hours before pMCAO had more severe injury than mice that had bone fracture 24 hours before pMCAO. Our data showed that bone fracture shortly before stroke also increases neuroinflammation and exacerbates ischemic cerebral injury. Our findings suggest that inhibition of neuroinflammation or management of stroke risk factors before major bone surgery would be beneficial for patients who are likely to suffer from stroke.

## Introduction

Bone fracture is a common health problem that can cause long-term disability. After adjusting for competing risk of death, the residual lifetime risk of fracture for women and men from age 60 is 44% and 25%, respectively [1]. Stroke and bone fracture also share some common risk factors, such as hypertension and diabetes mellitus [2,3]. Stroke is one of the most devastating complications for bone fracture patients. Although the incidence of stroke after bone fracture is rare, bone fracture patients with post-fracture stroke have poor functional recovery and require more care the first year after bone fracture than those who do not have stroke [4].

The treatment options for stroke patients with fractures are limited. Currently, intravenous thrombolysis is widely accepted and is still the only therapy approved by the U.S. Food and Drug Administration for the management of acute ischemic stroke [5,6]. For most of the individuals who have not received thrombolysis, antiplatelet or anticoagulation therapy is recommended to decrease the incidence of recurrent stroke [5,7]. However, thrombolysis or anticoagulation therapies can increase the incidence of fracture hemorrhage. There are no guidelines on the use of antithrombotic drugs after fracture [8]. Understanding the impact of bone fracture on stroke recovery and the mechanisms will help in developing new preventive and therapeutic strategies to improve patients’ outcomes.

We have shown that bone fracture 1 day after ischemic stroke in mice exacerbates neuronal injury and behavioral deficits, which are associated with an increase in neuroinflammation and oxidative stress [9]. Actvation of α-7 nicotinic acetylcoline receptor reduces neuroinflammation and ischemic brain injury [10].

In this study, we tested the hypothesis that bone fracture shortly before stroke exacerbates neuroinflammation and ischemic cerebral injury in mice.

## Materials and Methods

### Ethics Statement

Animal experimental procedures were approved by the Institutional Animal Care and Use Committee (IACUC) at the University of California, San Francisco, and conformed to National Institutes of Health guidelines. Mice were fed standard rodent food and water ad libitum, and were housed (maximum of 5 per cage) in sawdust-lined cages in an air-conditioned environment with 12-hour light/dark cycles. Animal husbandry was provided by the staff of the IACUC under the guidance of supervisors who are certified Animal Technologists, and by the staff of the Animal Core Facility. Veterinary care was provided by IACUC faculty members and veterinary residents located on the San Francisco General Hospital campus.

For surgeries, mice were anesthetized with 2% isoflurane inhalation, and given acetaminophen with drinking water one day before and one day after surgical procedures. Buprenorphine (0.1 mg/kg body weight) was injected intra-peritoneally to reduce pain: the first dose at the beginning of the procedure, the second dose 4–6 hours later, and then every 8–12 hours as needed. An animal's ability to drink water, feed, ambulate, guard painful areas, and its general overall appearance were evaluated five minutes after surgery. Animals were placed in their home cages when they were able to ambulate, feed, and drink unaided. They were monitored twice a day on the first day, daily during the first week, and then 3 times a week until they were euthanized.

The monitor criteria included: (1) wound inflammation diagnosed by swelling, edema or dehiscence; (2) wound bleeding diagnosed by local enlargement; (3) pain diagnosed by body movement and gesture; (4) neurological defects, abnormal gait, hemiplegia, or coma; (5) body weight loss; and (6) foot necrosis.

For all cases of wound enlargement, inflammation, bleeding or pain, veterinary staff was consulted to determine whether the animal should be treated or euthanized. The staff’s instructions were followed as to the kind and dose of antibiotics to be used for the particular infection. If the animals were not responsive to treatment, or if there was any evidence of post-operative respiratory compromise or bleeding, they were euthanized. In our experiments, none of the mice became so severely ill that they needed to be removed before the experiments ended.

The following methods were used to euthanize the mice at the end of the study. For the histological study, mice were perfused with 4% paraformaldehyde through the left cardiac ventricle after being anesthetized through inhalation of 2% isoflurane. For the protein analysis, mice were perfused with 1X PBS through the left cardiac ventricle after being anesthetized with 2% isoflurane inhalation. Brain samples were collected after paraformaldehyde or PBS perfusion.

### Animals

**Fig 1:**
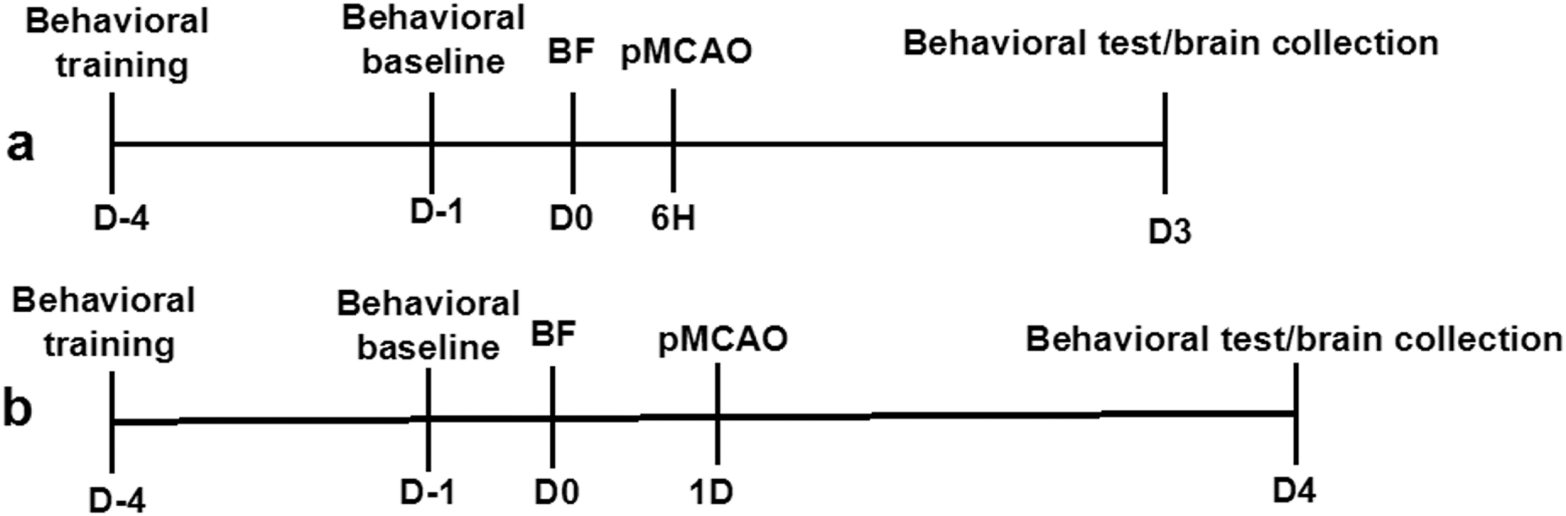
Experimental design. Time course for group with bone fracture (A) 6 hours before pMCAO, and (B) 24 hours before pMCAO. All mice underwent behavioral training 4 days before bone fracture, and baseline behavior performance was collected 1 day before bone fracture. Bone fracture was performed at day 0 (D0). Behavioral tests were performed 3 days after pMCAO. Brain samples were collected after the behavioral test. TF: tibia fracture.

C57BL/6J male mice (WT) were purchased from the Jackson Laboratory (Bar Harbor, ME, USA). Ccr2/^RFP/+^/Cx3cr1/^GFP/+^ transgenic mice that have red fluorescent protein gene knocked into one allele of C-C motif Chemokine receptor-2 (Ccr2^RFP/+^) and green fluorescent protein gene knocked into one allele of Cx3cr1 (Cx3cr1^GFP/+^) mice [11] were provided by Israel F. Charo at the University of California, San Francisco. Fifty-six 10 to 12-week-old WT mice and 30 10 to 12-week-old Ccr2/^RFP/+^/Cx3cr1^GFP/+^ mice were randomly assigned to 5 groups (Table 1). Fig 1 shows the experimental design. Researchers, blinded to the group assignment, performed neurobehavioral tests, infarct volume measurement, and cell counting.

**Table 1:** Experimental groups.

Group	Surgery	Sacrifice day	# of mice group (WT/Ccr2^RFP/+^/Cx3cr1/^GFP/+^)
1	BF	3d(control of #4)	10 / 6
2	BF	4d(control of #5)	10 / 6
3	Stroke	3d	12 / 6
4	BF-6h stroke	3d	12 / 6
5	BF-24h stroke	4d	12 / 6

BF: tibia fracture; BF-6h stroke: pMCAO was performed 6 hours after tibia fracture; BF-24h stroke; pMCAO was performed 24 hours after tibia fracture; Sacrifice day: days after tibia fracture.

### Tibia Fracture Surgery

Tibia fracture was performed on mice under aseptic surgical conditions using the method described previously [9]. After anesthetizing the mice with 2% isoflurane inhalation, the right hind limb with an intramedullary fixation was fractured. Animals were allowed to recover spontaneously from anesthesia under warm conditions. Rectal temperature was maintained at 37±0.5°C using a thermal blanket throughout the surgical procedure. Two doses of buprenorphine (0.1 mg/kg of body weight) were injected intraperitoneally at the beginning of the surgery and 4 hours later.

### Permanent Distal Middle Cerebral Artery Occlusion (pMCAO) Model

Six or 24 hours after tibia fracture, the left distal middle cerebral artery was permanently occluded (pMCAO) using procedures described below. Mice were anesthetized with 2% isoflurane inhalation. A 10-mm skin incision was made from the left orbit to the ear, followed by a 2 mm^2^ craniotomy under aseptic surgical conditions. The middle cerebral artery was then permanently occluded using electrical coagulation just proximal to the pyriform branch [9,12]. Body temperature was maintained at 37±0.5°C using a thermal blanket during surgery. A laser Doppler flow-meter (Vasamedics, Little Canada, MN, USA) was used to monitor the surface cerebral blood flow to ensure the success of the MCAO procedure. Mice with surface cerebral blood flow in the ischemic core >15% of the baseline or had massive bleeding because of the arterial injuries were excluded from the experiment. In this study, 6 mice were excluded from experiments and replaced by additional mice. Two doses of buprenorphine (0.1 mg/kg of body weight) were injected intraperitoneally at the beginning of the surgery and 4 hours after. Mice were allowed to recover from anesthesia under warm conditions.

### Behavioral Tests

Adhesive Removal Test was performed to assess potential somatosensory neglect [13]. Briefly, a piece of adhesive tape (0.3x0.3 cm) was placed on one of the forepaws, and the time the mouse took to remove the tape was recorded. The maximum testing time was 120 seconds (s). Mice were trained twice daily for 4 days before the bone fracture procedure to obtain an optimal level of performance. The adhesive removal times were recorded after 2 practice trials 1 day before bone fracture (D-1, baseline), and 3 days after stroke (Fig 1). It took longer for the mice to remove the tape from the paw on the opposite side of the stroke. Since the infarct in our model was on the left side of the brain, the adhesive removal times from the right paw were more relevant.

Corner Test was performed to detect sensorimotor and postural asymmetries after ischemic stroke [14]. Mice were placed between two 30 x 20 cm boards. Both sides of their vibrissae were stimulated as they approached the corner. The mice would then move up and turn to face the open end. For normal mice, the frequency of right and left turns would be equal. The stroke mice could not sense the stimulation on the stroke side, and hence, they made more turns to the ipsilateral side of the lesion (to the left in this study). Three different sets of 10 trials were conducted. Turning not incorporated in a rearing movement was excluded.

### Infarct Volume Estimation

Cresyl violet staining was used to determine infarct volume. Three days after pMCAO, mice were perfused with 4% paraformaldehyde and brain samples were then collected. A series of 20-μm-thick coronal sections were made, of which 1 in 10 (200 μm apart) was stained with cresyl violet and imaged. The infarct areas were outlined and their pixel areas were quantified using IMAGE J (National Institutes of Health, Bethesda, MD, USA). The infarct volumes were estimated by multiplying the sum of infarct areas from all cresyl violet-stained sections by 200 μm [10].

### Immunohistochemistry

Immunohistochemical staining was performed using a series of 20-μm-thick sections. All the quantifications were performed using the sections in the same anatomical region (bregma 1.2–1.4 mm). Sections were incubated with the following primary antibodies: CD68 (a macrophage marker, 1:50, AbD Serotec, MCA1957, Raleigh, NC, USA), and neuronal nuclei (NeuN, 1:500, MAB377, Millipore, Bedford, MA, USA) at 4°C overnight. After washing with phosphate-buffered saline (PBS), sections were then incubated with Alexa Fluor 647-conjugated, Alexa Fluor 594-conjugated, and Alexa Fluor 488-conjugated IgG (1:500, Invitrogen, Carlsbad, CA, USA). Negative controls were performed by omitting the primary or the secondary antibodies. Terminal deoxynucleotidyl transferase-mediated deoxyuridine triphosphate (dUTP) nick end-labeling (TUNEL) assay was performed using the dedicated kit (ApopTag, Millipore) following the manufacturer’s instructions. The co-localization of NeuN-TUNEL staining was verified with confocal imaging. TUNEL positive neurons (NeuN positive) at the peri-infarct region inside the cortical infarct border were quantified using image J (NIH, USA) on three different pictures per mice taken under 40X objective.

CD68^+^, Ccr2^+^, and Cx3cr1^+^ cells, as well as Ccr2^+^ and Cx3cr1^+^ double-positive cells, were counted separately using 3 different pictures per mice taken under 40× objective at the peri-infarct region outside the infarct border [15].

### Brain Protein Extraction and Enzyme-linked Immunosorbent Assay (ELISA)

After being anesthetized through isoflurane inhalation, mice were perfused with 1X PBS through the left ventricle to remove blood. Brain samples were collected and frozen in dry ice. Peri-infarct brain regions were dissected out and homogenized in 1X cell lysis buffer (Cell Signaling, Danvers, MA, USA), supplemented with 1 mM PMSF (Cell signaling). The lysates were centrifuged at 13,000 rpm for 25 minutes, and the supernatants, collected. Protein concentrations were measured using the Bradford Protein Assay (Bio-Rad Laboratories, Hercules, CA, USA). IL-1ß (R&D systems, Minneapolis, MN, USA) and IL-6 (R&D systems) levels were measured (using 312 μg protein from each animal) through ELISA assays following the manufacturer’s instructions.

### Statistical Analyses

Data are presented as mean ± SD. Sample size for each test is indicated in the figure legends. All analyses in this study, except ELISA data, were performed using SPSS 16.0 software (SPSS Inc, Chicago, IL, USA). Gaussian distribution was tested with d’Agostino and Pearson omnibus normality test. Equalities of variances were tested with the F test. For multiple comparisons, means were compared using one-way ANOVA followed by Fisher’s least significant difference (LSD) test. A two-tailed P value < 0.05 was considered statistically significant. ELISA data were analyzed with GraphPad Prism 6 software using the One-Way ANOVA analysis with multiple comparisons.

## Results

### Bone Fracture Before pMCAO Enhanced Brain Injury

**Fig 2:**
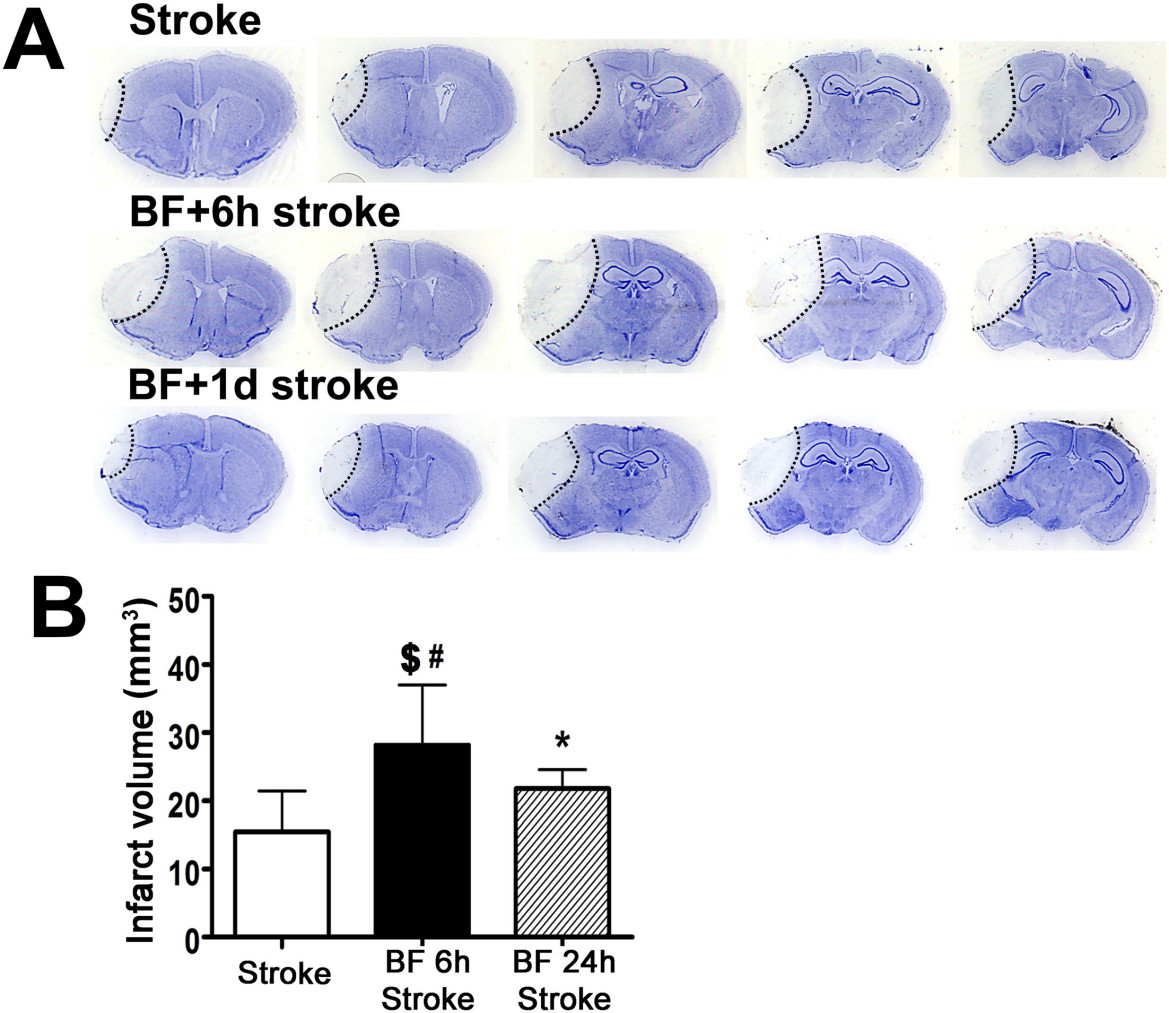
Bone fracture before pMCAO increased infarct volume. (A) Representative images of cresyl violet-stained brain sections. Scale bar: 1mm μm. (B) Quantification of infarct volume. *: P = 0.05 compared to stroke group, $: P<0.001: compared to stroke group; #: P<0.05, compared to bone fracture 24 hours before pMCAO group. n = 10 in stroke and tibia fracture 6 hours before stroke group. n = 9 in tibia fracture 24 hours before stroke group. BF 6h Stroke: tibia fracture 6 hours before pMCAO; BF 24h Stroke: tibia fracture 24 hours before pMCAO.

We found in our previous study that bone fracture one day after ischemic stroke exacerbates brain injury [9,12]. To determine if bone fracture before stroke has any influence on ischemic brain injury, we performed tibia fracture on mice 6 or 24 hours before pMCAO and analyzed infarct volume and neuron apoptosis. Compared to mice subjected to pMCAO (15.4±5.99 mm^3^) only, infarct volume increased in mice subjected to bone fracture before pMCAO (Fig 2). Tibia fracture 6 hours before stroke was 28.1±8.89 mm^3^ (p<0.001); tibia fracture 24 hours before stroke was 21.8±2.82 mm^3^ (p< 0.05). The infarct volumes in mice that received pMCAO 6 hours after bone fracture were also larger than those that received pMCAO 24 hours after (p<0.05).

**Fig 3:**
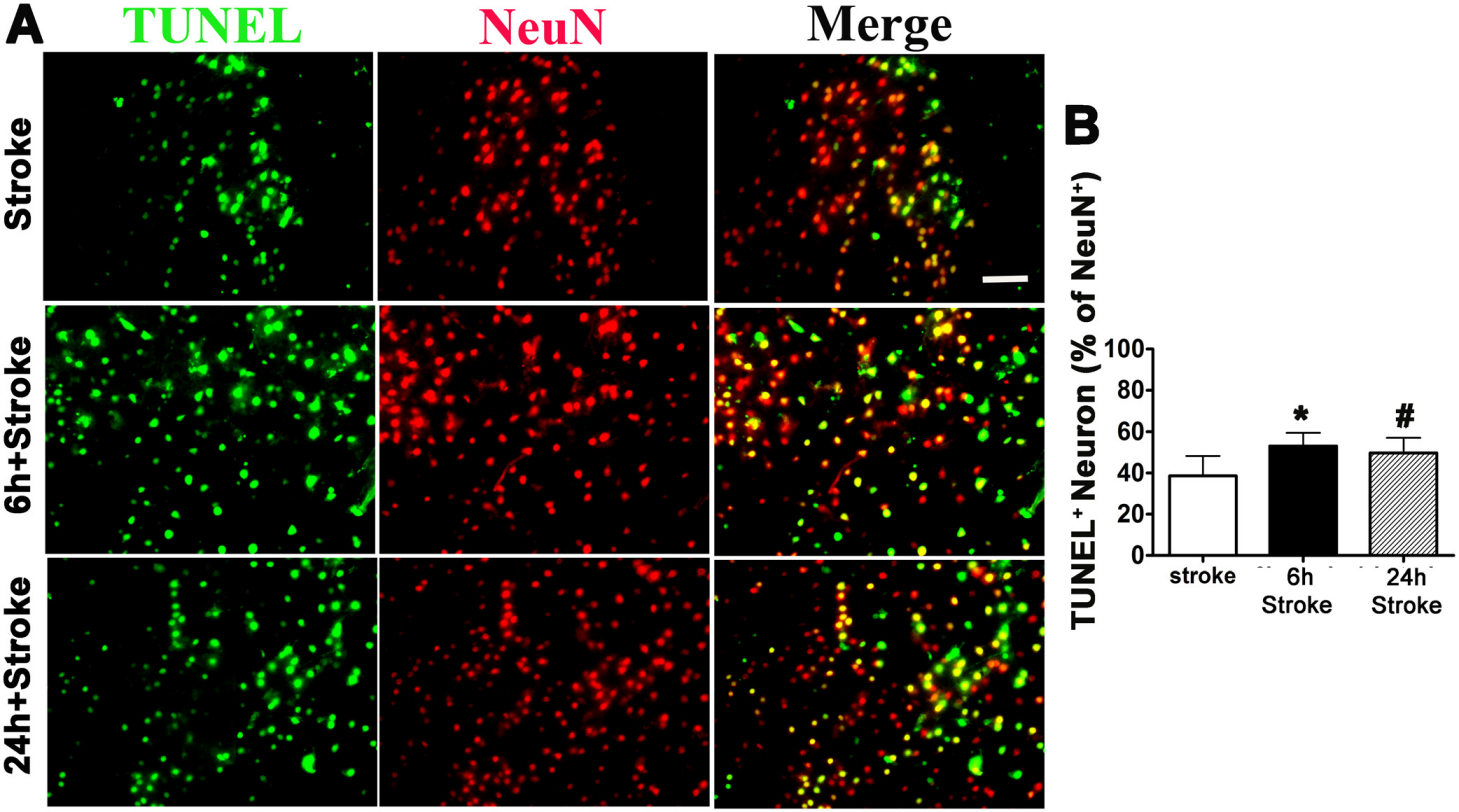
Bone fracture increased apoptotic neurons in the peri-infarct region. (A) Representative images of TUNEL and NeuN antibody-stained sections. Scale bar: 50 μm. (B) Quantification of TUNEL positive neurons. * P = 0.007 compared to stroke-only group; #: P = 0.027 compared to stroke-only group n = 6. 6h Stroke: tibia fracture 6 hours before pMCAO; tibia fracture 24 hours before pMCAO.

Bone fracture also increased TUNEL positive neurons in the peri-infarct region: 53.1±6.51% neurons in mice that received bone fracture 6-hours before pMCAO (p = 0.007), and 49.8±7.33% in mice that received bone fracture 24 hours before stroke (p = 0.027, Fig 3), compared to that of stroke-only mice (38.6±9.65, Fig 3). Therefore, bone fracture before stroke increased ischemic brain injury.

### Bone Fracture Before pMCAO Increased Behavioral Deficits

**Fig 4:**
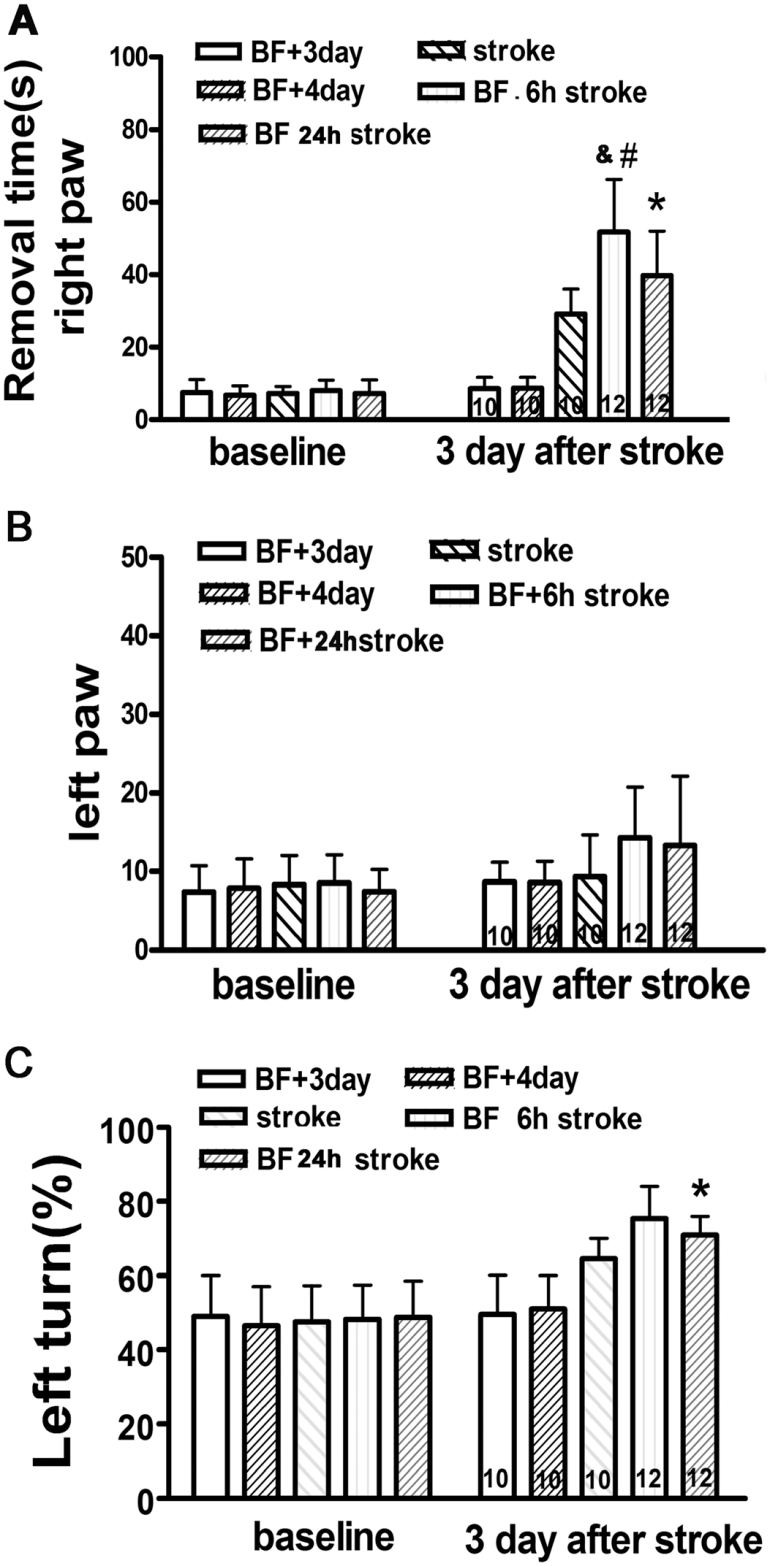
Bone fracture before pMCAO increased behavioral deficits. (A) Adhesive removal test (right paw). Mice that received bone fracture took longer to remove adhesive from right paw. #: P<0.001 compared to stroke-only mice. *: P = 0.05: compared to stroke-only group. & = 0.02 compared to mice that received tibia fracture 24 hours before pMCAO. (B) Adhesive removal test (left paw). n = 10 for control groups (BF+3-day, BF+4day) that received tibia fracture and sham pMCAO, and had their behavioral test done 3 or 4 days after bone fracture. (C) Corner test. #: P = 0.001 compared to stroke group; *:P = 0.035 compared to stroke group. n = 10 for control groups (BF+3-day, BF+4day) that received tibia fracture and sham pMCAO, and had their behavioral test done 3 or 4 days after bone fracture. n = 10 for pMCAO-only group. n = 12 for groups that received bone fracture 6 hours or 24 hours before pMCAO. BF 6h Stroke: tibia fracture 6 hours before pMCAO; BF 24h stroke: tibia fracture 24 hours before pMCAO.

To test if bone fracture before pMCAO increases behavioral dysfunction, we performed adhesive removal and corner tests on mice 3 days after pMCAO. To match the tibia fracture time, we included two groups of mice that were subjected to tibia fracture only in the tests. One group had behavioral tests before and 3 days after the fracture, which was used as control for the group subjected to pMCAO 6 hours after tibia fracture. The other group was tested before and 4 days after tibia fracture, which was used as control of the group subjected to pMCAO 24 hours after tibia fracture. We found that compared to stroke-only mice (29±6.8 seconds), those subjected to bone fracture and pMCAO took longer to remove the sticker from the right forepaw (Fig 4A): bone fracture 6 hours before pMCAO group, 52±14.5 seconds, p<0.001; bone fracture 24 hours before pMCAO, 40±12.2 seconds, p = 0.05. The mice subjected to bone fracture 6 hours before pMCAO also took a longer time than those that received bone fracture 24 hours before pMCAO (p = 0.02). The time it took to remove the adhesive from the left paw was similar among all the groups (p>0.05, Fig 4B).

Compared to stroke-only mice (65±5.5%), mice with bone fracture and stroke made more left turns (lesion side) in the corner test: bone fracture 6 hours before pMCAO group, 75±8.7% (p = 0.001); bone fracture 24 hours before pMCAO group, 71±5.2% (p = 0.035, Fig 4C). Therefore, bone fracture shortly before ischemic stroke exacerbated stroke-related behavioral dysfunction.

### Bone Fracture Before pMCAO Increased Microglia/ Macrophage Infiltration in the Peri-infarct Region

**Fig 5:**
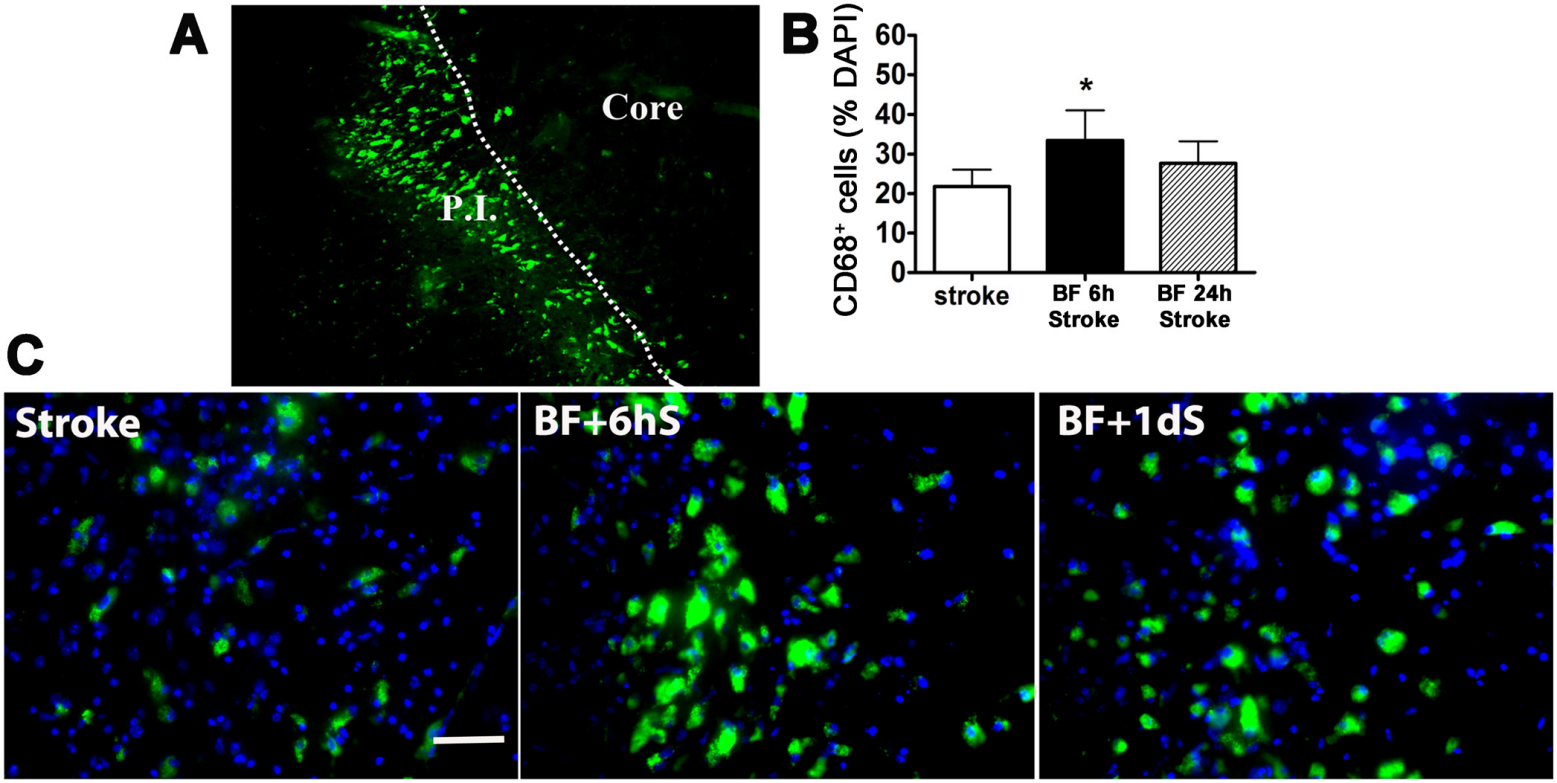
Bone fracture increased CD68^+^ macrophages in the peri-infarct region. (A) Image illustrates infarct core (Core), infarct border (dotted line) and the peri-infarct region (P.I.). (B) Quantification of CD68^+^ cells. *: P = 0.004, compared to stroke-only group. (C) Representative images of anti-CD68 antibody-stained sections. BF + 6hS: mice that received tibia fracture 6 hours before pMCAO; BF+1dS: mice that received tibia fracture 24 hours before pMCAO. Scale bars: 50μm. N = 6.

To analyze if bone fracture before stroke increases neuroinflammation, we quantified CD68^+^ cells in the peri-infarct cortex (Fig 5A). We also used CCR2^RFP/+^CX3CR1^GFP/+^ mice to identify bone marrow-derived macrophages and local microglia. We found that bone fracture 6 hours before stroke increased the number of CD68^+^ cells in the peri-infarct regions (33.4±7.62% of total DAPI positive nuclei) compared to the stroke-only group (21.80±4.27%, p = 0.004, Fig 5B & 5C).

**Fig 6:**
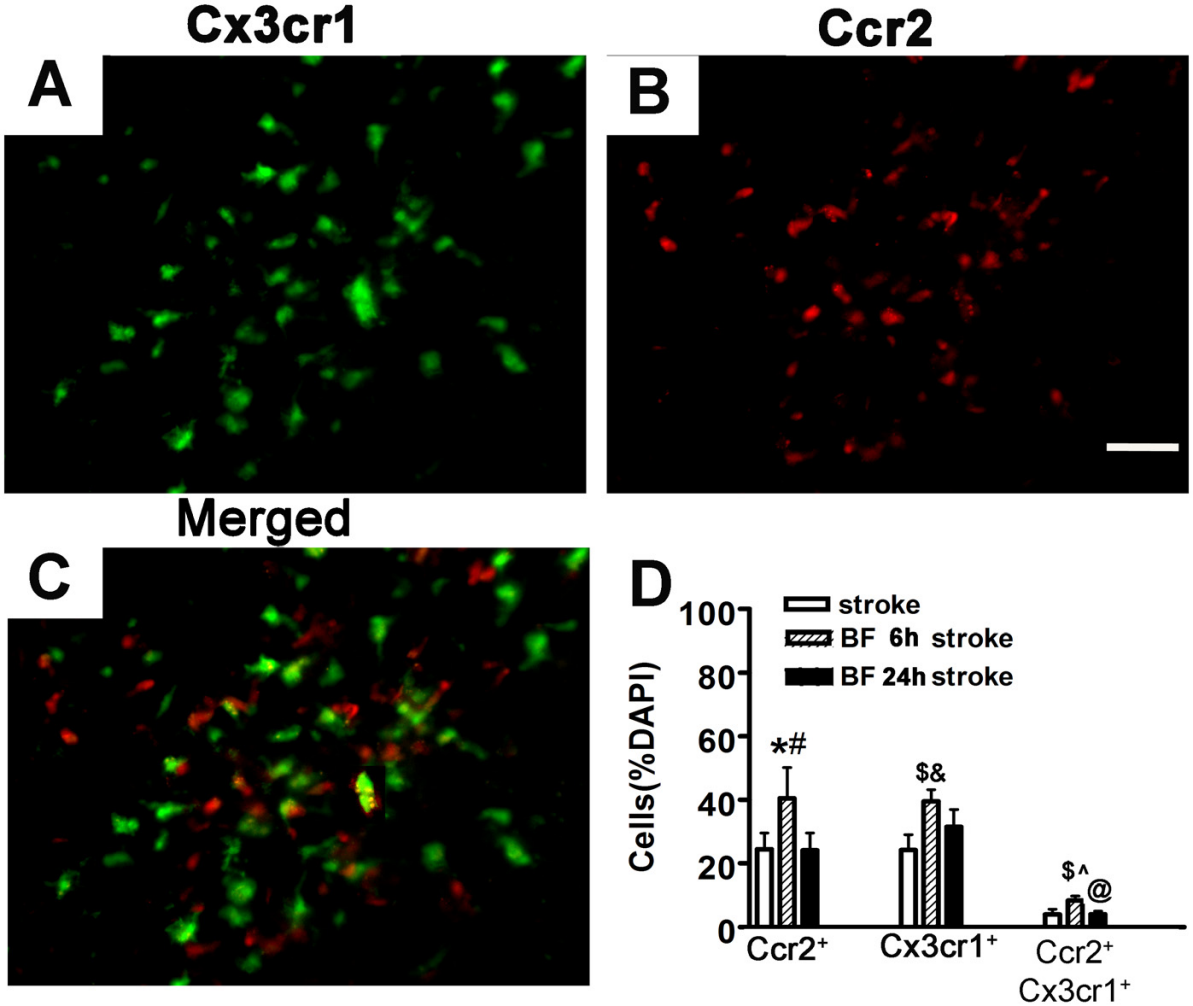
Bone fracture increased both microglia and bone marrow-derived macrophages in the peri-infarct region. (A-C) Representative image of Cx3cr1 positive cells (green, A), Ccr2 positive cells (red, B), and Cx3cr1 and Ccr2, double positive cells (yellow, C). (D) Quantification of Cx3cr1^+^, Ccr2^+^ and Ccr2 and Cx3cr1 double positive cells in the peri-infarct region. *: P = 0.001, compared to stroke-only group, #: P = 0.001, compared to mice that received tibia fracture 24-hours before pMCAO. $: P<0.001 compared to stroke-only group, &: P = 0.01 compared to bone fracture 24 hours before pMCAO group. ^: P<0.001 compared to bone fracture 24-hours before pMCAO group. BF 6h Stroke: tibia fracture 6 hours before pMCAO; BF 24h stroke: tibia fracture 24 hours before pMCAO. Scale bar: 50μm.

Mice that had bone fracture 6-hours before pMCAO also had more bone marrow-derived macrophages (40.4±9.78% of DAPI labeled nuclei, CCR2^+^) in the peri-infarct regions compared to stroke-only mice (24.4±5.15%, p = 0.001) and bone fracture 24 hours before pMCAO mice (24.0±5.57%, p = 0.001, Fig 6B & 6D). Mice that received bone fracture 6 hours before pMCAO also had more microglia (39.6±3.66%, CX3CR1^+^) in the peri-infarct regions compared to stroke-only mice (24.2±4.88%, p <0.001, Fig 6A & 6D) and mice that received bone fracture 24 hours before pMCAO (31.4±5.54%, p = 0.01, Fig 6D).

In addition, mice that received bone fracture 6 hours before pMCAO had more CCR2/CX3CR1 double positive cells (8.4±1.40%) compared to those that received pMCAO only (4.0±1.68%, p < 0.001, Fig 6C & 6D) or mice that received bone fracture 24 hours before pMCAO (4.0±0.99%, p<0.001, Fig 6C & 6D). These data suggest that bone fracture shortly before stroke increases both bone marrow-derived macrophages and microglia in the peri-infarct region; some of the bone marrow-derived macrophages acquired the microglia phenotype.

### Bone Fracture Before pMCAO Did Not Increase Interleukin 1ß (IL-1 ß) and IL-6 in the Peri-infarct Region

**Fig 7:**
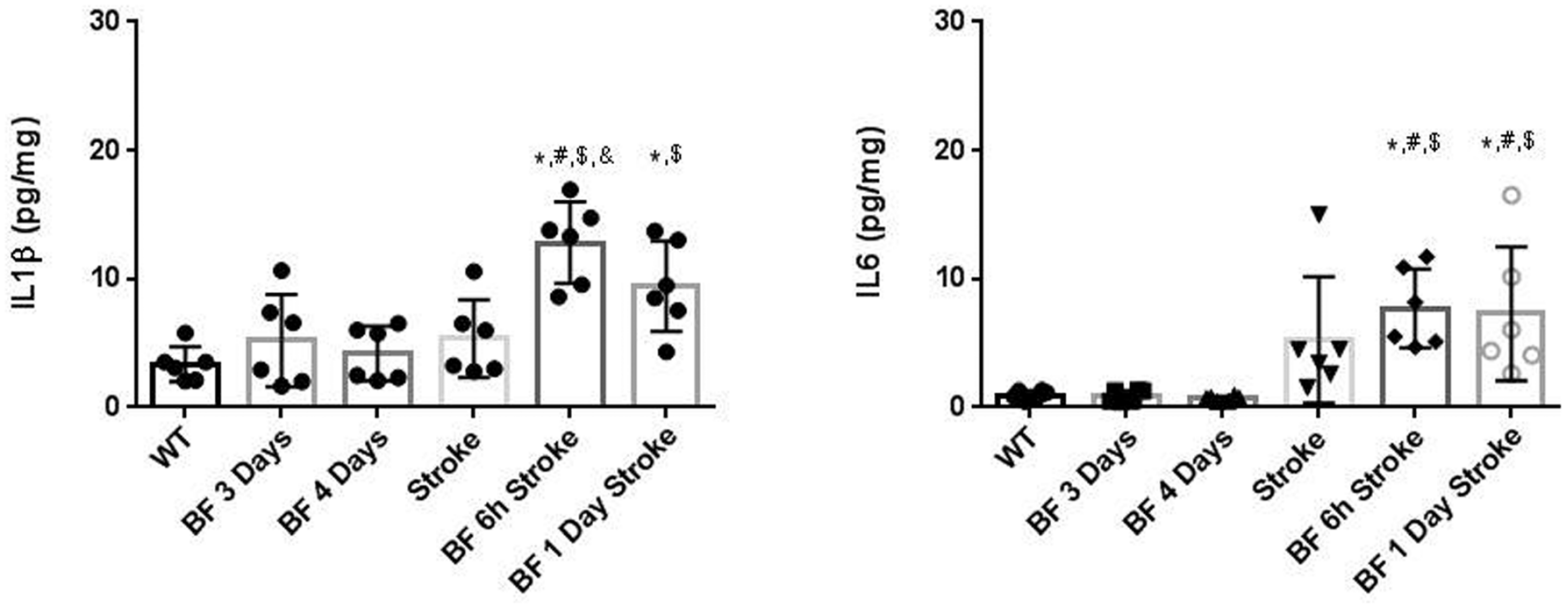
IL1ß and IL-6 levels increased in the peri-infarct brain of stroke mice. (A) Quantification of IL-1ß expression. *: P<0.05 vs. WT group; #: P = 0.001 vs. BF 3-day group; $: P<0.001 vs. BF 4-day group; &: P<0.01. (B) Quantification of IL-6 expression. *: P<0.05 vs. WT group; #: P<0.05 vs. BF 3-day group; $: P<0.05 vs. BF 4-day group. N = 6. BF 3 Days: Samples were collected 3 days after tibia fracture, which served as control for mice subjected to tibia fracture 6 hours before pMCAO. BF 4 Days: Samples were collected 4 days after tibia fracture, which served as control for mice subjected to tibia fracture 24 hours before pMCAO; BF 6h Stroke: tibia fracture 6 hours before pMCAO; BF 1 Day Stroke: tibia fracture 24 hours before pMCAO.

Previous studies have shown that tibia fracture increases interleukin 1ß (IL-1 ß), IL-6, and tumor necrosis factor α (TNFα) in the blood and hippocampus. It would be interesting to determine whether these cytokine levels are higher in the peri-infarct region of mice subjected to tibia fracture plus stroke vs. mice subjected to stroke only. TNF α expression increases in plasma shortly after tibia fracture, peaks at 30 minutes and returns to normal 12 hours later [16]. In this study, the brain samples were collected 3 days (group with tibia fracture 6 hours before the stroke group) or 4 days (group with tibia fracture 24 hours before stroke) after tibia fracture. It is unlikely to detect bone fracture induced increase of TNFα expression at these time points. The increase of IL-1ß and IL-6 lasts longer [17,18]. Therefore, we analyzed IL1-ß and IL-6 levels in the peri-infarct tissues. We found that the IL-1ß level was significantly higher in mice with both tibia fracture and stroke than in uninjured mice (WT) and mice with stroke alone. The IL-1ß in mice that have been subjected to tibia fracture 6 hours before stroke was also higher than in mice with tibia fracture or stroke only (Fig 7A). IL-6 levels were higher in mice subjected to both injuries than in WT and mice subjected to tibia fracture alone (Fig 7B).

## Discussion

In this study, we examined the effects of bone fracture shortly before stroke on stroke-related brain injury and functional deficits. Two bone fracture time-points, 6 hours and 24 hours before stroke, were analyzed. We found that bone fracture before stroke exacerbates ischemic cerebral injury and behavior dysfunction and neuroinflammation. Bone fracture 6 hours before stroke had a more negative impact on ischemic injury than bone fracture 24 hours before pMCAO.

Tibia fracture increases systemic and hippocampal inflammation [16,19], and we reported previously that tibia fracture 1 day after ischemic stroke exacerbates neuroinflammation and injury [9]. Clinically, patients with post-fracture stroke have poor functional recovery and require more care the first year after bone fracture than those who did not have stroke [4]. The impact of bone fracture occurring before stroke has not been studied.

We showed in this study that bone fracture occurring shortly before stroke also increases ischemic brain injury. Compared to mice that had bone fracture 24 hours before stroke, those with bone fracture 6 hours before had more severe neuroinflammation and brain injuries. Terrando et al showed that serum TNFα peaks at 30 minutes and HMGB1, IL-1ß, IL-6 at 6 hours after tibia fracture [16,18]. Therefore, we included a group that had bone fracture 6 hours before stroke. The severity of brain injury in this group compared to the group that received bone fracture 24 hours before stroke could be due to the higher inflammatory cytokines in the blood.

Inflammation plays an important role in stroke pathology and has a biphasic effect [20]. At the acute stage of stroke, it has an adverse effect on stroke recovery [20–22], and modulating inflammation has been shown to promote the healing process and functional recovery [9,23,24]. Both experimental and clinical evidence suggests that augmented inflammation triggered by bone fracture is responsible for the negative impact of bone fracture on stroke recovery. (1) In mice, tibia fracture increases pro-inflammatory cytokines, alarmins and monocytes/macrophages in the blood and the hippocampus [16,17]. (2) Inflammation induces acute cognitive/behavioral dysfunction [16,25,26]. (3) Increased tumor necrosis factor α (TNFα) in the blood of mice with tibia fracture [16] modulates synaptic plasticity and neural injury [27,28]. (4) Prolonged systemic inflammation increases functional impairments after focal ischemia in rats [29]. (5) Our previous study showed that activation of anti-inflammation α-7 nicotinic acetylcholine receptor reduces inflammation and improves functional recovery in mice suffering from stroke or stroke followed by tibia fracture 1 day later [10].

In this study, we determined that bone fracture 6 hours before stroke increased CD68^+^ macrophages, the levels of IL-1ß and IL-6 in the peri-infarct region, which was accompanied by increased neuronal death and behavior dysfunction. Using Ccr2^RFP/+^Cx3cr1^GFP/+^ mice, we showed that both Ccr2^+^ bone marrow-derived macrophages and Cx3cr1^+^ microglia increased and that some of bone marrow-derived macrophages adopted the microglia phenotype expressing Cx3cr1. Whether inhibition of bone marrow-derived macrophages homing to the infarct region at the acute stage could reduce neuronal death and improve functional recovery of mice with both bone fracture and stroke needs to be studied in the future.

In addition to microglia and bone marrow-derived macrophages, astrocytes are also capable of secreting inflammatory factors (e.g., cytokines, chemokines and nitric oxide) that increase adhesive molecules in the endothelial surface and facilitate the infiltration of leukocytes and macrophages [30–33]. In addition, astrocytes normally prevent excitotoxic glutamate elevations in brain extracellular space. The astrocyte glutamate uptake is reduced in the ischemic brain, resulting in glutamate effluxes that enhance oxidative stress, neuroinflammation and neuronal death. The role of astrocytes in the neuroinflammation of mice subjected to stroke and tibia fracture will be studied in the future.

In summary, there is currently no established therapeutic strategy to prevent the occurrence of stroke in bone surgery patients who are likely to suffer from stroke. Using a mouse model of bone fracture before pMCAO, we demonstrated that bone fracture shortly before pMCAO exacerbates ischemic cerebral injury, behavior dysfunction and neuroinflammation. In our previous animal study, we showed that depletion of macrophages by clodrolip, inhibition of high-mobility-group box chromosomal protein-1 (HMGB1) through a specific antibody [9], or reduction of inflammation through activation of α7 nicotinic acetylcholine receptor [10] reduce stroke injury of mice that were subjected to tibia fracture one day after stroke. Therefore, block-specific inflammatory pathways could be utilized in developing a strategy specifically to prevent stroke in bone surgery patients who are likely to suffer from it post-surgery and improve functional outcomes in patients suffering from both injuries.
