# Correction: Bone Fracture Pre-Ischemic Stroke Exacerbates Ischemic Cerebral Injury in Mice

**DOI:** 10.1371/journal.pone.0164214

**Published:** 2016-09-29

**Authors:** Liang Wang, Shuai Kang, Dingquan Zou, Lei Zhan, Zhengxi Li, Wan Zhu, Hua Su

The second affiliation for the third author is not indicated. Dingquan Zou is also affiliated with:

Department of Anesthesiology, Second Xiangya Hospital, Central South University, Changsha, Huna, China.
